# Disease Acceptance and Eudemonic Well-Being Among Adults With Physical Disabilities: The Mediator Effect of Meaning in Life

**DOI:** 10.3389/fpsyg.2020.525560

**Published:** 2020-10-22

**Authors:** Małgorzata Szcześniak, Agata H. Świątek, Małgorzata Cieślak, Daria Świdurska

**Affiliations:** Institute of Psychology, University of Szczecin, Szczecin, Poland

**Keywords:** disease acceptance, eudemonic well-being, meaning in life, adults, mediation

## Abstract

The acceptance of disability is recognized as one of the most frequently mentioned factors that plays a particularly significant role in subjective well-being. However, so far, only a very small amount of research has been undertaken to clarify how and why acceptance of illness relates to eudemonic well-being. Hence, comprehension of the direct and indirect effects underlying this relationship seems essential for interventions that increase the recovery of people with impairments and enhance their quality of life. The current research was aimed at investigating the association between acceptance of illness, meaning in life, and eudemonic well-being, as well as the possible mediatory effect of meaning in life on the relationship between acceptance of illness and well-being. The sample consisted of 102 participants (71% women) aged between 20 and 64 years. The respondents had a range of different impairments (e.g., cerebral palsy, neurological disorders, spinal muscular atrophy, and sight defects). The Acceptance of Illness Scale, the Meaning in Life Questionnaire, and the Ryff Scales of Psychological Well-Being were used. It was confirmed that acceptance of illness correlated positively and significantly with the presence of meaning, self-acceptance, positive relations, environmental mastery, personal growth, general well-being, cohesion, flexibility, communication, and family satisfaction. The presence of meaning mediated the relationship between acceptance of illness and general well-being with its four other dimensions: self-acceptance, environmental mastery, purpose in life, and personal growth. Conversely, the search for meaning did not have any mediatory effect on this relationship.

## Introduction

Disability, whether genetic or accidental, traumatic or progressive, is a growing problem area in public health, and a large social concern (Keany and Glueckauf, 1993; Tough et al., 2017). According to the World Report on Disability (World Health Organization [WHO] and World Bank, 2011), nearly 200 million people out of one billion diagnosed with some kind of disability experience serious difficulties in functioning due to their long-term physical, psychological, or intellectual impairments. In much of the available empirical research, chronic conditions of disease or disability pose a critical health threat, lower social participation rates, and tend to reduce patients’ life satisfaction (Nosek et al., 1995; Tough et al., 2017).

In spite of the negative experience, dysfunction, and pathology related to different forms of disabilities (Elliott et al., 2002; Reuman et al., 2013), a number of empirical studies have demonstrated that the chronically ill and persons with disabilities have the potential for psychological growth and well-being (Cordova et al., 2001; Reuman et al., 2013; Kim et al., 2016; Wettstein et al., 2016). For instance, it has been found that emotional competence (Rey et al., 2013), social support (Ryan et al., 2007), task-oriented coping (Jones et al., 2003), goal reengagement (Van Bost et al., 2019), redefined life purpose (Pakenham, 2007), engagement in volunteering (Fekete et al., 2019), and disease acceptance (Ditchman et al., 2017; Rosińczuk and Kołtuniuk, 2017) are essential correlates or predictors of subjective well-being among individuals with a chronic physical or psychological disability.

Since a very small amount of research has been performed so far to clarify how illness acceptance relates to well-being (Brown et al., 1981; Okely and Gale, 2016), the current research was aimed at investigating the association between both acceptance of disease and eudemonic well-being of people with physical disabilities. The concept of acceptance of disability has been defined as the acknowledgment of, and adaptation to, a disability (Martz et al., 2000), acceptance of loss and value change (Keany and Glueckauf, 1993), and awareness that infirmity has occurred and is real (Carver et al., 1989). Zhang et al. (2019) noticed that acceptance of one’s own infirmity denotes the degree to which patients shape their knowledge by integrating their lifestyle into the experience of dealing with the disability. The acceptance of disability is an important aspect that clarifies why some people adjust to their disability and others do not (Park, 2019). In this vein, Verhoof et al. (2014) found that the perceptions of illness of young adults with a childhood-onset somatic condition influenced their emotional well-being. Moreover, disability acceptance has been recognized as one of the most frequently mentioned factors that plays a particularly significant role in well-being (Ditchman et al., 2017) and adjustment to a new way of life (Kowalska et al., 2019). In contrast, poorer acceptance of the disease and its shortcomings were associated with the occurrence of acute symptoms of depression and lower assessment of quality of life (Rosińczuk and Kołtuniuk, 2017). Acceptance of the limitations imposed by a long-lasting disease and readjustment of life goals had a constructive influence on well-being in adolescents and young adults (Casier et al., 2011) and lowered negative emotions or reactions related to the disease and its treatment (Cybulski et al., 2017). Therefore, on the basis of previous research, it can be assumed that acceptance of disability may be an indicator of eudemonic well-being that, as opposed to hedonic well-being, emphases more existential concerns (Wood and Joseph, 2010) which seem to be inherent to the acceptance of disability and meaning in life (Ososkie and Holzbauer, 2004). In this approach, eudemonic well-being consists of more than the satisfaction, pleasure, and happiness that characterize hedonistic well-being. Instead, it refers to a valuable, flourishing, and fulfilling life (Ryan and Deci, 2001) which encompasses autonomy, personal growth, self-acceptance, life purpose, mastery, and positive relatedness (Ryff and Keyes, 1995).

**FIGURE 1:**
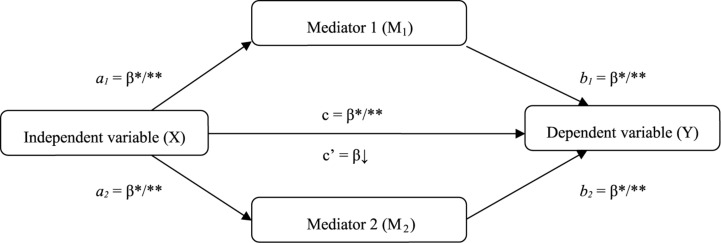
Theoretical model of the role of meaning in life, and the relationship between acceptance of illness and well-being. ^∗^*p* < 0.05; ^∗∗^*p* < 0.01.

As a second goal, we wanted to examine, through mediation analysis, the indirect effects underlying the relationship between disease acceptance (independent variable) and eudemonic well-being (dependent variable), since comprehension of other factors that may interact with acceptance and well-being seem essential for interventions that increase the recovery of people with impairments and enhance their quality of life (Figure 1). We aimed to verify why acceptance of disability may be an indicator of eudemonic well-being.

Among the most frequently mentioned psychological factors that may play a decisive role in accepting a chronic disease and maintaining well-being in the context of a long-lasting disability is having meaning in life (Mpofu et al., 2017). In fact, scholars have long emphasized that the experience of and longing for meaning are crucial in human life (Heintzelman and King, 2014). Meaning in life is a conceptually complex construct (Hooker et al., 2018) and can be understood from different perspectives (Negri et al., 2020). For example, Frankl (2009) considered it the main motivational force in a person’s life. Other authors (Peterson et al., 2005) defined a meaningful life as a life in which people consider themselves connected to something larger than the self. According to Steger et al. (2009), the presence of meaning (Mediator 1, Figure 1) refers to the degree to which individuals grasp, make sense of, and see significance in their lives. People who have discovered meaning in life perceive their existence as significant and purposeful. In turn, the search for meaning (Mediator 2, Figure 1) denotes the active pursuit and efforts to establish or increase comprehension of purpose of life (Steger et al., 2008). While the presence of meaning represents the result or outcome of previously undertaken attempts to gain sense, the searching reflects an active and continuous process of looking for meaning (Dezutter et al., 2013).

With regard to the independent variable and its relationship with meaning in life, Piekutin et al. (2018) pointed out that life with a chronic disease is frequently associated with the need for the person to not only accept their condition, but also to find their inner resources and reformulate their goals. According to Park and Folkman’s (1997) stress-coping theoretical framework, dealing with negative events and adverse life conditions (e.g., crisis, disease, or death) can be an important meaning-making process through which people might cope with stressful circumstances. Having purpose and direction has been related to positive adjustment among stroke survivors (Affleck et al., 1987; Chow et al., 2007). The perception of life as significant, purposeful, and valuable allows people to bear, transcend, and survive the direst situations (Frankl, 2009).

With respect to the dependent variable and its association with meaning in life, empirical research has shown consistent and positive associations of the presence of meaning with many measures of well-being and a very wide variety of other indicators (Steger et al., 2011). According to some theories (Ryff and Singer, 1998), meaning in life is one of the main characteristics of well-being. People can be thought to be experiencing well-being if they feel their lives are meaningful and experience a sense of purpose. Several studies have related meaning in life with positive functioning (Wood and Joseph, 2010) and positive emotions (Ryff and Keyes, 1995). Conversely, a lack or diminution of meaning in life has been associated with depression, anxiety, and distress (Debats et al., 1993).

While the relationship between the presence of meaning and well-being seems clear, the association between the search for meaning and well-being is less well-defined (Steger, 2012) and somehow inconsistent. For example, Dezutter et al. (2013) found that the search for meaning was negatively related to feelings of optimism, acceptance, and life satisfaction. Similarly, Park et al. (2010) noted that the search for meaning in general was also negatively associated. However, the same authors mentioned that it was positively linked to greater life satisfaction, more happiness, and less depression among those participants who had already developed substantial meaning in their lives. In other studies, the search for meaning was not significantly related to life satisfaction (Newman et al., 2017).

With regard to the potentially mediating role of meaning in life in the relationship between illness acceptance and eudemonic well-being, it has been confirmed that meaning provides people with the sense that their lives matter (Steger, 2012) even when these lives are filled with disease. The reason for this mediating effect may be due to the fact that people have an inclination to look for sense, as it is their vital need and intuitive drive (Frankl, 2009). Discovering and achieving meaning helps individuals to cope with suffering and distress. Meaning in life is formed from uncertainty, struggle, and disease but, at the same time, it serves as a fundamental component of people’s psychological and subjective well-being (Steger et al., 2013; Khumalo et al., 2014). Experiencing meaning in life helps patients confronted with a chronic illness to maintain higher levels of well-being (Dezutter et al., 2013). More precisely, meaning seems to be related with higher psychological well-being among individuals living with spinal cord injuries (deRoon-Cassini et al., 2009), brain tumors (Ownsworth and Nash, 2015), institutionalized and community-residing older adults (Reker, 1998), chronically ill patients (Dezutter et al., 2013), and patients in palliative care settings (Boston et al., 2011). In other studies (Zhou and Xu, 2018), meaning in life mediated the relationship between self-acceptance and psychological well-being in a cohort of gastrointestinal cancer patients. Thompson et al. (2003) observed that, in the context of chronic or life-threatening illness and disability, a sense of meaning in life has an important function in psychological adjustment. People who can deal with a crisis and find meaning in adversity are more adaptable and report higher well-being (Shao et al., 2014). Boyraz et al. (2015) found that participants who considered death as part of life disclosed having a higher sense of meaning in their lives, which, in turn, predicted grief symptoms. Meaning in life seems to motivate people to persevere rather than to quit in face of difficult conditions, and to engage with stressful or traumatic events in an adaptable rather than inflexible manner (McKnight and Kashdan, 2009).

Given this background, it was hypothesized that the desire to find or have meaning in chronic disease would be a natural motivational drive for a better life. Thus, the following assumptions were made:

*Hypothesis 1 (H1)*: There is a positive correlation between the acceptance of illness, the presence of meaning, the search for meaning in life, and dimensions of well-being.

*Hypothesis 2 (H2)*: Meaning in life, expressed by searching and presence, mediates the relationship between acceptance of illness and dimensions of well-being.

## Materials and Methods

### Participants

The sample consisted of 102 participants (71% women) aged between 20 and 64 years. The average age was almost 32 (*M* = 31.86; *SD* = 11.03). The respondents had a range of different impairments related mainly to physical conditions. The biggest group was formed by respondents with cerebral palsy (23%), followed by motor impairment (21%), a neurological disorder (14%), spinal muscular atrophy (9%), sight defects (5%), muscular dystrophy (4%), myelitis (4%), myelomeningocele (4%), paraparesis (3%), arthrogryposis multiplex congenita (3%), multiple sclerosis (2%), epilepsy (2%), Parkinson’s disease (2%), paralysis of the lower limbs (2%), bilateral hearing loss (2%), and paraplegia (2%).

### Procedure

The participants were recruited through social network services for people with physical disabilities. The Facebook group administrators were asked whether our online battery of questionnaires could be posted on their groups’ pages. The invitation was directed to persons with impairments mainly associated with permanent disorders of the development of movement and posture, muscle weakness, or problems with vision, sensation, balance, or the motor system. All respondents who decided to take part in the study were given general information about the research aims and were prompted with a written informed consent form. Only after providing their agreement the participants were invited to fill in the questionnaires. The protocol was approved by the Bioethics Committee of the Institute of Psychology at the University of Szczecin and was conducted in accordance with the Declaration of Helsinki.

### Measures

#### Assessment of the Acceptance of Illness Scale

The Acceptance of Illness Scale (AIS; Felton and Revenson, 1984), in the Polish adaptation by Juczyński (2009), consists of eight statements which measure the degree of acceptance of limitations due to illness, as well as the feeling of dependence imposed by illness. The participants are asked to rate each statement on a scale from 1 to 5, where 1 denotes *strongly agree*, and 5 denotes *strongly disagree*. The acceptance of illness is measured against the total number of points each participant obtains (8–40 points). A low score is considered to be less than 20 points, and values above 30 points indicate a high level of acceptance of one’s disease. An average score ranges from 20 to 30 points (Kowalczyk et al., 2019). Therefore, the lower the score, the less the patient tolerates their illness. The reliability, measured using Cronbach’s alpha coefficient, in the current study was high at 0.89, which demonstrates the good psychometric properties of the tool.

#### Assessment of the Meaning in Life Questionnaire

The Meaning in Life Questionnaire (MLQ; Steger et al., 2006), in the Polish adaptation by Kossakowska et al. (2013), is a 10-item scale that measures the presence and the search for meaning in one’s being and existence. The participants evaluate each item on a 7-point Likert scale ranging from 1 (*strongly disagree*) to 7 (*strongly agree*). The items are summed to yield a total score of meaning. The higher the final score, the more intense is the presence and the search for meaning. The original study (Steger et al., 2006) reports a good coefficient alpha of 0.86 for presence of meaning and 0.87 for search for meaning. In the current survey, the Cronbach’s alpha was 0.87 for presence of meaning and 0.77 for search for meaning, demonstrating good internal consistency for both subscales.

#### Assessment of the Ryff Scales of Psychological Well-Being

The Ryff Scales of Psychological Well-Being (PWB; Ryff and Keyes, 1995), in the Polish adaptation by Karaś and Cieciuch (2017), consists of 18 items to measure six intercorrelated components of well-being: (1) “self-acceptance” refers to positive evaluations of oneself and one’s past life, (2) “positive relations with others” signifies the possession of quality relations with others, (3) “autonomy” relates to a sense of self-determination, (4) “environmental mastery” represents the capacity to successfully handle one’s life and the surrounding world, (5) “purpose in life” denotes the belief that one’s life is purposeful and meaningful, and (6) “personal growth” indicates a sense of continued development as a person. Each dimension is operationalized by means of a 3-item scale. Respondents rate each item according to a six-point agreement scale from 1 (*strongly disagree*) to 6 (*strongly agree*). Higher scores indicate greater well-being on each component considered. In the present study, the internal consistency of the whole PWB was 0.79. The values of Cronbach’s alpha for all of the dimensions of well-being were as follows: self-acceptance (α = 0.61), positive relations (α = 0.56), autonomy (α = 0.50), environmental mastery (α = 0.64), purpose in life (α = 0.32), and personal growth (α = 0.43). Although the internal consistency coefficients for purpose and growth are lower than the other subscales, they are higher than those reported by Ryff and Keyes (1995), van Dierendonck (2004), Fernandes et al. (2010), and Novo et al. (1997). Moreover, since Cronbach’s alpha values are sensitive to the number of items, we checked the mean inter-item correlation for the items. In fact, inter-correlation between all of the factors of the PWB was 0.508, higher than the ideal range for the inter-item correlation of 0.2 to 0.4 suggested by Briggs and Cheek (1986).

### Statistical Analysis

For the analyses of the statistical data, the Statistical Package for the Social Sciences (SPSS software version 23, IBM Corporation) was used. The data distribution was tested by means of skewness and kurtosis (Morgan and Griego, 1998). Descriptive statistics were calculated, and Pearson’s correlation coefficients (*r*) for parametric data were computed.

The statistical *a priori* power for the current study was conducted using G^∗^Power 3.1.9.4 (Heinrich-Heine-Universität, Düsseldorf, Germany) to reduce the risk of an underpowered result (Faul et al., 2009). We applied the *t* test for the correlation, taking into consideration the recommended higher power criteria of 0.95, and a significance criterion α of 0.05. Although there is mixed empirical evidence about the relationship between the variables considered in our study, some analyses illustrate that there is a positive, albeit modest (*r* = 0.40) (Kozieł et al., 2016) or even strong (*r* = 0.59) in magnitude, association between acceptance of illness and quality of life. Likewise, the associations between the different aspects of meaning in life and psychological well-being vary across the studies from small (*r* = 0.20) to large (*r* = 0.58) correlations (Shek, 1992). On the basis of the previous literature, we expected a medium effect of *r* = 0.35. The G^∗^Power analysis with these input parameters indicated that the total sample size would require a minimum of 79 respondents.

A linear regression analysis was performed to test the normality of the residuals and constant variance (homoscedasticity), multicollinearity, outliers, and to verify if and by how much age, sex, and the type of disease would act as potential confounders in the model. Mahalanobis’ distance and Cook’s distance were computed. Although in many studies there were no statistically significant differences in acceptance of illness with regard to gender (Staniszewska et al., 2017; Kowalska et al., 2019), there were some results that showed the mean values of acceptance of illness being lower in males (Jankowska-Polańska et al., 2016) or in females (Kazimierska-Zając et al., 2018). Furthermore, other researchers (Jankowska-Polańska et al., 2016; Sierakowska et al., 2017; Janiszewska et al., 2019) found that the level of acceptance of illness was significantly reduced with age. Finally, some studies have shown that the type of disease can act as a determining factor of life quality and acceptance of illness (Chabowski et al., 2019). The potential confounders were entered at Step 1. All variables hypothesized as predictors of well-being were entered at Step 2.

The PROCESS macro for SPSS was run to establish the extent to which acceptance of illness influenced well-being through meaning in life. For the present analysis, the 95% confidence interval of the indirect effects was calculated using 5000 bootstrapped resamples.

## Results

### Descriptive Statistics

Acceptance of illness, presence of meaning and search for meaning, self-acceptance, positive relations, autonomy, environmental mastery, purpose in life, personal growth, and general well-being were screened for skewness and kurtosis to evaluate the normality of the scale’s distribution. We assumed indices less than the ± 2 commonly considered acceptable limits of a normal distribution (Gravetter and Wallnau, 2014; George and Mallery, 2016). No variables exceeded the cutoffs of ± 2 (Table 1).

**TABLE 1:** Descriptive statistics for the AIS, MLQ, and PWB (*N* = 102).

Scales	M	SD	Skewness	Kurtosis
1. AI	24.90	8.89	0.005	–1.252
2. SM	26.04	4.68	–0.688	0.365
3. PM	24.07	4.89	–0.363	0.162
4. SA	12.33	3.51	–0.359	–0.671
5. PR	14.01	3.32	–0.562	–0.593
6. AU	13.17	3.14	–0.386	–0.427
7. EM	13.31	3.36	–0.279	–0.925
8. PL	12.55	3.44	–0.282	–0.549
9. PG	14.58	2.74	–0.808	–0.019
10. GW	79.99	12.95	–0.305	–0.686

*AI, acceptance of illness; SM, search for meaning; PM, presence of meaning; SA, self-acceptance; PR, positive relations; AU, autonomy; EM, environmental mastery; PL, purpose in life; PG, personal growth; GW, general well-being.*

### Testing for the Normality of Residuals, Multicollinearity, and Confounders

**FIGURE 2:**
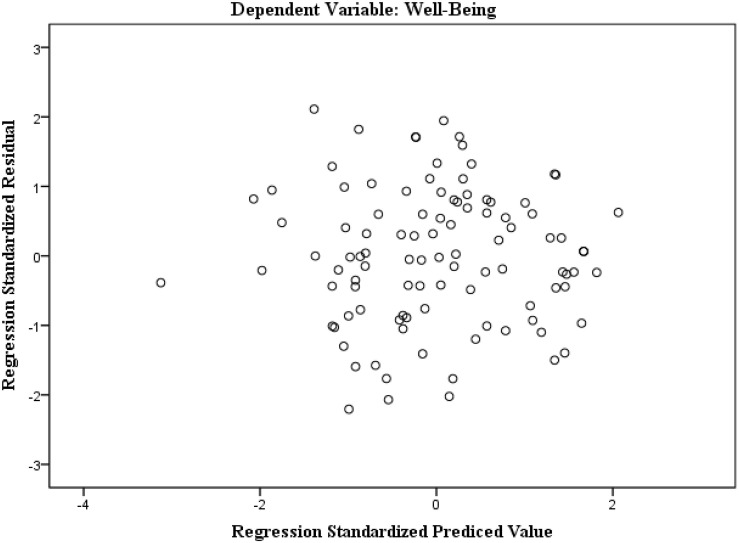
The scatterplot of the standardized residuals against standardized predicted values.

In terms of the assumptions associated with the residuals, the homoscedasticity and autocorrelation were evaluated. A scatterplot of standardized residuals against the predicted values (Figure 2) shows that the plotted points had no obvious pattern or systematic structure, being evenly divided above and below their mean value of zero. The graphical presentation was confirmed by the Shapiro–Wilk test. The results of *p* = 0.76 for both unstandardized and standardized residuals indicated no variations from normality. The Durbin–Watson statistic was 1.93, close enough to the value 2, thus proving that the residuals were independent of each other.

In respect to multicollinearity (Mansfield and Helms, 1982), the tolerance values ranged from 0.878 to 0.981, above the critical value of 0.1 suggested by Field (2000) as a considerable collinearity problem. The variance inflation factor (VIF) values ranged from 1.019 to 1.139, not exceeding the commonly assumed threshold of 5.0 (Hair et al., 2017). Both results suggest that multicollinearity was unlikely to be an issue in our study. Mahalanobis’ distance procedure was conducted, using the chi-square distribution with a probability estimate for a case being an outlier (*p* < 0.001) (Fidell and Tabachnick, 2003). No cases were identified as probable multivariate outliers since the lowest *p*-value in the sample was 0.00130. Moreover, Cook’s value (between 0.000 and 0.087) was under the point at which the researcher should be concerned (less than 1) (Fidell and Tabachnick, 2003). Hierarchical regression analyses also showed that neither sex, age, nor type of disease made a significant unique contribution to the model, explaining only 1% of the variance (*R*^2^ = 0.012): sex (β = 0.022, *t* = 0.216, *p* = 0.829), age (β = −0.102, *t* = −1.014, *p* = 0.313), and type of disease (β = −0.024, *t* = −0.231, *p* = 0.818). The predictors explained an additional 62% of the variance, even after controlling for the effects of potential confounders (sex, age, and type of disease).

### Correlations Between the Study Variables

Hypothesis H1 (Table 2) was confirmed to a large extent as acceptance of illness correlated positively and significantly with presence of meaning, self-acceptance, positive relations, environmental mastery, personal growth, general well-being, cohesion, flexibility, communication, and family satisfaction. Moreover, search for meaning was positively and significantly associated with the presence of meaning and well-being and all of its dimensions. The presence of meaning was positively and significantly linked to well-being and all of its dimensions, except for autonomy.

**TABLE 2:** Correlations between dimensions of AIS, MLQ, and PWB (*N* = 102).

Variables	AI	SM	PM	SA	PR	AU	EM	PL	PG	GW
1. AI										
2. SM	0.15									
3. PM	0.42***	0.28**								
4. SA	0.62***	0.37***	0.46***							
5. PR	0.42***	0.31**	0.28**	0.41***						
6. AU	0.11	0.27**	0.15	0.32**	0.023*					
7. EM	0.76***	0.21*	0.45***	0.68***	0.48***	0.19^t^				
8. PL	0.12	0.23*	0.30**	0.09	0.27**	–0.03	0.16^t^			
9. PG	0.35***	0.36***	0.33***	0.53***	0.36***	0.38***	0.44***	0.40***		
10. GW	0.61***	0.44***	0.50***	0.77***	0.70***	0.51***	0.75***	0.48***	0.76***	

*p < 0.05; **p < 0.01; ***p < 0.001. ^*t*^tendency; AI, acceptance of illness; SM, search for meaning; PM, presence of meaning; SA, self-acceptance; PR, positive relations; AU, autonomy; EM, environmental mastery; PL, purpose in life; PG, personal growth; GW, general well-being.

### Mediations

In the following part of the analyses (H2), only presence of meaning was introduced as a potential mediator between the independent variable (acceptance of illness) and the dependent variable (well-being), since searching of meaning did not meet the conditions for the mediation analysis because of a lack of correlation with acceptance of illness. The PROCESS macro for SPSS (Table 3) showed that the c path (the direct effect) reduced in magnitude after the introduction of presence of meaning in five models out of seven (c’ path). On the basis of the gained outcomes, it can be stated that presence of meaning mediates the relationship between acceptance of illness and general well-being with its four other dimensions: self-acceptance, environmental mastery, purpose in life, and personal growth. Conversely, search for meaning did not have any mediatory effect on this relationship. Therefore, hypothesis H2 was partially confirmed.

**TABLE 3:** Significant outcomes of mediation analyses from acceptance of illness to well-being assessing indirect effects of presence of meaning (*N* = 102).

Model	a path	b path	c path	c’ path	Indirect effect	B(SE)	Lower CI	Upper CI
1. AI – PM – SA	0.23***	0.17**	0.24***	0.20***	0.0402	0.0140	0.0143	0.0695
2. AI – PM – EM	0.23***	0.10*	0.29***	0.26***	0.0243	0.0115	0.0043	0.0497
3. AI – PM – PL	0.23***	0.21**	0.04(*ni*)	−0.01(*ni*)	0.0501	0.0187	0.0174	0.0908
4. AI – PM – PG	0.23***	13*	0.11***	0.08*	0.0298	0.0133	0.0050	0.0581
5. AI – PM – GW	0.23***	0.79***	0.89***	0.71***	0.1847	0.0607	0.0811	0.3196

*p < 0.05; **p < 0.01; ***p < 0.001. ni, non-significant; AI, acceptance of illness; PM, presence of meaning; SA, self-acceptance; EM, environmental mastery; PL, purpose in life; PG, personal growth; GW, general well-being.

## Discussion

The current research aimed at investigating the association between acceptance of illness, meaning in life, and well-being (H1), as well as the possible mediatory effect of meaning in life on the relationship between acceptance of illness and well-being (H2).

Firstly, the positive correlation between acceptance of illness and presence of meaning in life is consistent with earlier studies. For example, Pinquart et al. (2009) showed that adaptation to a chronic illness and disability required patients to reevaluate their life goals and expectations. Krok and Telka (2018) found that presence of meaning was negatively correlated with perceiving illness as a threat, obstacle/loss, and harm. Conversely, presence of meaning was positively associated with feeling an illness as a benefit or challenge, or having value and importance.

Dezutter et al. (2013) reported that people who perceive their life as meaningful tend to cope better with medical challenges. The lack of an association between acceptance of illness and search for meaning found in the present study is similar to the results obtained by some other researchers. Krok and Telka (2018) observed that there were no statistically significant relations between search for meaning and illness perception among cancer patients.

In terms of the presence of meaning in life and well-being, the present research also confirmed, to a large extent, the outcomes received by other researchers. It has been affirmed that experiencing meaning in life appears to be related to psychosocial adjustment among individuals adapting to the demands of disease (Park et al., 2010; Steger et al., 2011; Sherman and Simonton, 2012). Likewise, Dezutter et al. (2013) found that chronically ill patients who experienced high levels of meaning had higher levels of well-being compared with those patients who declared lower levels of meaning. When it comes to the relationship between the search for meaning and well-being, our outcomes did not confirm those previous findings that suggest a lack of or negative correlations between the search for meaning and well-being (Yek et al., 2017). For example, Park et al. (2010) provided evidence that the search for meaning was negatively associated with well-being. Moreover, Damasio and Koller (2015) observed a negative correlation between searching for meaning and both satisfaction with life and subjective happiness. Still, Park et al. (2010) noted that the search for meaning was positively related to well-being only among those respondents who had already demonstrated a considerable sense of life. Kossakowska et al. (2013) also found that searching for meaning was positively, although weakly, associated with life satisfaction and its affirmation. Such a positive relationship could be explained by referring to the original connotation given to the concept of search for meaning. Initially, searching for meaning was recognized as a positive construct, irrespective of whether the person was going through a difficult situation or not (Damasio and Koller (2015). Moreover, Steger et al. (2006, 2011) observed that the presence of meaning does not impede the search for further meaning, and people who proactively look for sense feel confident about giving meaning to their experience. Likewise, Reker (2000) acknowledged that the search for meaning can be considered to be a life-affirmation and deficit-based motivation. This would explain why the obtained results show a positive relationship between the search for meaning and all of the dimensions of well-being.

Finally, hypothesis 2 was partially confirmed since only in five out of seven models did the presence of meaning in life act as a mediator in the relationship between acceptance of illness and well-being/its dimensions. At the same time, search for meaning had a mediatory effect on this relationship in none of the seven models. These outcomes deserve special attention since they seem to confirm that both components of meaning, although sharing some aspects in common (Bakracheva, 2019), are independent (Steger et al., 2011) and may play different roles in the context of acceptance of illness and psychological adaptability (Kossakowska et al., 2013). More precisely, our findings show that the acceptance of illness is related to increased well-being when people who struggle with chronic disease alter their viewpoint on life, reconsider their values and priorities, give meaning to their health condition, and have a sense of continuous growth and development as a person (Staniszewska et al., 2017). Once individuals encounter and have a sense of meaning, they deal with disease in a more meaningful way, which can lead to higher well-being. In fact, Dezutter et al. (2013) observed that patients experiencing higher levels of meaning in life had greater levels of well-being compared to those patients who experienced lower levels of meaning. The same cannot be said about search for meaning, which in our study did not mediate the relationship between disease acceptance and well-being. When people with a disability do not have meaning in life, the process of searching can be more challenging and disappointing (Park et al., 2010). They can feel dissatisfied with themselves and perceive little control over their life (Steger et al., 2008).

Another interesting finding obtained in the current study concerns the mediatory role of the presence of meaning in the relationship between the acceptance of illness and general well-being with its four other dimensions: self-acceptance, environmental mastery, purpose in life, and personal growth. These results confirm theoretical premises presented by Steger et al. (2008) who implied that people who have presence of meaning better understand themselves and the world around them. In other words, our results may suggest that people with a physical disability who accept their condition may also acknowledge and assume multiple aspects of the self, including the disability and all of its consequences. Likewise, such people may display competence in managing their environment by choosing or creating a context suitable to their personal needs. Moreover, the presence of meaning is not indifferent to beliefs that having goals and a sense of direction make life purposeful. Finally, people who are aware of the personal growth process seem better equipped to handle challenges related to health struggles and display higher initiative when going through a crisis related to their disability (Borowa et al., 2018). Therefore, on the basis of the current study, it can be assumed that adjustment to a prolonged condition of impairment leads to higher well-being when people revise their life goals through the experience of meaning more than through engagement in achieving meaning in life.

### Limitations

The present study highlights the mediatory effect of meaning in life on the association between acceptance of illness and well-being among adults with a disability. However, a few weaknesses are important to take note of. The first limitation is that the cross-sectional nature of the data set does not allow for the formation of causal links among the variables. Longitudinal and experimental studies would yield a more detailed and rigorous illustration of the relationships tested. Secondly, because of the uneven proportion of female to male participants in the present study, a guarded interpretation is recommended when relating the outcomes to the gender differences. It is suggested that, in future research, a similar study should be implemented with a greater proportion of men. Thirdly, since the variables were assessed through self-reported measurement methods, the outcomes could be influenced by socially desirable responding (SDR). In future studies, researchers might adopt questionnaires projected to monitor each participant’s tendency toward SDR. Next, we did not gather more information on disability severity, length, and level of impairment. Including these variables in the analysis, along with the age and gender, could provide new information in understanding the dynamics of the relationship between the factors studied. Moreover, the research was conducted with the use of online social services. This detail is important to keep in mind since access to Facebook and other Internet social networks is limited to younger and technologically skilled participants. There is a need to back up digital research with more traditional methods, providing a larger group of respondents with the opportunity to participate. Finally, we assumed that meaning in life would mediate the relationship between illness acceptance and eudemonic well-being. However, it would be interesting to verify whether this relationship is not bidirectional, that is, whether meaning in life is not an independent variable and illness acceptance acts as a potential mediator. In fact, some studies (Jaworski et al., 2018; Chen et al., 2019; Janiszewska et al., 2019) have confirmed that a proper attitude toward illness may be of particular importance in the context of giving meaning to disease and may lead to effective functioning.

## Conclusion

In terms of the theoretical implications, the current research broadens our understanding of the interplay between acceptance of illness, meaning in life, and well-being among adults with a physical disability, since it is one of only a few pieces of research to have explored this issue. More specifically, it yields important confirmations of the mediatory role of experiencing meaning in life between the adjustment to a chronic illness and eudemonic well-being and its dimensions. The outcomes confirm that finding meaning plays a motivating role during the difficult process of disease acceptance and experiencing well-being. At the same time, there is a question that can be raised in the context of the present study in regard to the lack of mediatory effects of the search for meaning. Moreover, our findings confirmed the search-to-presence model, which suggests that looking for meaning is related to presence of meaning (Steger et al., 2008). With respect to the clinical implications, the current outcomes suggest that patients’ perceptions of their lives as significant and purposeful can be a crucial psychological resource in maintaining their optimal well-being during a challenging time of disease.

## Data Availability Statement

The datasets generated for this study are available on request to the corresponding author.

## Ethics Statement

The studies involving human participants were reviewed and approved by The Bioethics Committee of the Institute of Psychology at the University of Szczecin. The patients/participants provided their written informed consent to participate in this study.

## Author Contributions

MS contributed to the ideation of the review and the search of literature, conducted the statistical analyses, and wrote the manuscript. AŚ contributed to the search of literature and wrote parts of the manuscript. MC contributed to the ideation of the review, collected data, and wrote parts of the manuscript. DŚ collected the data and wrote parts of the manuscript. All authors contributed to the article and approved the submitted version.

## Conflict of Interest

The authors declare that the research was conducted in the absence of any commercial or financial relationships that could be construed as a potential conflict of interest.
